# Clinicopathological and microbiological profile of osteomyelitis at a teaching hospital in India: A retrospective study

**DOI:** 10.6026/973206300220443

**Published:** 2026-01-31

**Authors:** Taruna Singh, Sakshi Chourasia, Suneel Kumar Ahirwar, Ramesh Agrawal

**Affiliations:** 1 Department of Microbiology, Shyam Shah Medical College, Rewa, Madhya Pradesh, India; 2 Department of Pathology, Government Medical College, Satna, Madhya Pradesh, India; 3 Department of Microbiology, MGM Medical College, Indore, Madhya Pradesh, India; 4 Department of Microbiology, Government Medical College, Satna, Madhya Pradesh, India

**Keywords:** Osteomyelitis, diabetes mellitus, *staphylococcus aureus*, chronic bone infection, histopathology, microbiology

## Abstract

Osteomyelitis, a complex bone infection, requires better management strategies to address its chronicity and microbial variations. Therefore, it is of interest to evaluate the demographic, clinical and microbiological characteristics of osteomyelitis cases. A retrospective analysis of 234 patients revealed a strong link between diabetes and chronic osteomyelitis. *staphylococcus aureus* was the most common pathogen identified. Histopathological findings showed chronic inflammation and bone necrosis in many cases. Early diagnosis and proper antimicrobial therapy are crucial in preventing chronic outcomes and improving clinical management.

## Background:

Osteomyelitis is a major infectious disease of the bones and bone marrow, it occurs in the form of an acute disease or a chronic and slow disease. The disease can become progressive when the diagnosis or treatment is late; thus, resulting in continuing bone destruction and possible long-term functional disability [1]. The infection can result due to hematogenous dissemination of the infection, direct transfer of microorganisms due to minor tissue foci, or direct introduction into the microorganism due to trauma or surgical practice. Chronic osteomyelitis is characterized as usually being characterized by persistent infection, devitalised bone, the formation of sequestrum, as well as inconsistent levels of reactive bone remodelling [2]. The epidemiology of osteomyelitis has changed over the last decades, as chronic forms are becoming more prevalent among adult populations. This change is to a great extent explained by the increased number of underlying comorbidities, including diabetes mellitus, peripheral vascular disease and illnesses connected to defective immunity [3]. In the case of diabetic foot infections in particular, diabetes mellitus is one of the key predisposing factors with deeper extension into the bone seriously increasing morbidity and risk of limb loss [4]. A number of studies have shown that diabetic foot ulcers are critical complications in which the involvement of the ossicles is of great concern, therefore, the need to identify them promptly and provide proper, timely management [5]. Microbiologically, *staphylococcus aureus* is still the most common causative agent in acute and chronic osteomyelitis. The pathogenicity of the organism is also increased by the fact that it is able to adhere to surfaces of bones, evade host defense systems and form biofilms, which contributes to its persistence and resistance to treatment [6]. The growing prevalence of methicillin-resistant *staphylococcus aureus* (MRSA) and other drug-resistant organisms further complicates empirical treatment, which is why culture-based identification and selection of the antibiotic should be of utmost priority on the basis of susceptibility [6]. Osteomyelitis diagnosis is based on a combined method of clinical examination, radiography, microorganism culture outcomes and histopathologic examination. Learning the clinicopathological and microbiological patterns of osteomyelitis in particular healthcare settings would be critical in maximizing patient care and providing effective empirical and definitive treatment approaches. Therefore, it is of interest to determine the clinicopathological and microbiological patterns of osteomyelitis in a healthcare setting, which is crucial for optimizing patient care and improving treatment outcomes.

## Materials and Methods:

It was a retrospective observational study conducted in a tertiary care teaching orthopedic department.

## Study population:

The hospital history of the patients with acute or chronic osteomyelitis within the stipulated period of the study was examined. All age groups and both sex patients with osteomyelitis confirmed clinically, by radiography and/or microbiological means were included. The exclusion criteria included cases that were not documented fully, those who were diagnosed only during the post-mortem examination and those who had been treated elsewhere in the healthcare centers before the case was presented. 234 patients who met the eligibility criteria were enrolled. This was deemed to be an adequate number to give a representative evaluation of the clinicopathological and microbiological features of osteomyelitis amongst the sample population. Medical records provided the relevant information on demographics, clinical characteristics, comorbidity, laboratory parameters, imaging, surgery and microbiological results. Special attention was paid to:

## Clinical features:

Symptoms duration, pain, swelling and fever and sinus tracts.

## Radiological findings:

Evaluation of plain radiograph and MRI to classify cases as acute or chronic osteomyelitis.

## Laboratory investigations:

Complete blood count, erythrocyte sedimentation rate (ESR), C-reactive protein (CRP) and other relevant biochemical tests.

Samples such as pus, bone biopsies and tissue taken during surgical debridement were cultured anaerobically and aerobically in standard microbiological methods. The automated identification systems were used to identify the pathogen and the process was supplemented by the traditional biochemical techniques where needed. The testing of antimicrobial susceptibility was done in compliance with Clinical and Laboratory Standards Institute (CLSI) recommendations. The bone and soft tissue specimens were fixed under 10% formalin, routinely processed and stained with hematoxylin and eosin. The microscopic examination was aimed at the inflammatory infiltrates, necrosis, fibrosis and granulation tissue to tell the difference between acute and chronic osteomyelitis. SPSS software version 26.0 was used to analyze the collected data and compile the data in Microsoft Excel. Continuous variables were described in terms of mean standard deviation or median interquartile range, whereas categorical variables were described in terms of frequency and percentages. The Chi-square test was used to determine associations of clinical parameters with microbiological results with statistical significance set at p-value <0.05.

## Results:

The sample size of this study was 234 patients who had osteomyelitis. The average age amounted to 39.6 years and 17.2 and most of the cases (40.2) were found in the category aged 31-50 years. There was also a clear male dominance as the study population was 65% men. The prevalence of associated comorbid conditions in the patients was 39.3 percent with diabetes mellitus the most common (25.6 percent), peripheral vascular disease (9.4 percent) and immunosuppression (4.3 percent). Patients (47.9 per cent) reported Symptoms longer than six weeks and 33.3 per cent of patients reported Sinus tract formation (Table 1). Statistically significant relationship between diabetes mellitus and type of osteomyelitis was found (Table 2). After comparing the non-diabetic and diabetic patients, it was found that chronic osteomyelitis was present in 70% of diabetic patients versus 58.6% of non-diabetic patients (p = 0.021) and diabetes mellitus was found to be a significant risk factor of chronic disease. Microbiological diagnosis revealed *staphylococcus aureus* as the major isolate (43.6%) and the next 15.4% of cases demonstrated methicillin-resistant *S. aureus* (MRSA). Other most frequently isolated would be the *Pseudomonas aeruginosa* (12%) and Gram-negative bacilli like *Escherichia coli* and *Klebsiella species* (13.6% combined). The proportion of *Streptococcus species* and polymicrobial infections were 6.8% and 5.1% of all cases, respectively and in 3.4% of samples the culture was sterile (Table 3). Examination of association between microbial isolates and type of disease showed a strong correlation between *S. aureus* (including MRSA) and chronic osteomyelitis (p = 0.034), which highlights its importance in chronic bone infection. Other organisms showed no statistically significant relationships (Table 4). These results indicated that 33.3% of patients had acute inflammatory changes on histopathological examination and 59 had features of chronic inflammation. The extent of severity and chronic nature of infection was revealed by necrotic bone in 47.9 percent, fibrosis and granulation tissue in 41.9 percent, formation of sequestrum in 30.8 percent and reactive new bone formation in 27.4 percent in a significant percentage of patients (Table 5).

## Discussion:

A high incidence of chronic osteomyelitis, frequent comorbidities and playback with *staphylococcus aureus* were apparent in this retrospective series of 234 patients as was previously described in the literature in explaining the intricate and long-standing nature of bone infections. The strong correlation between diabetes mellitus and chronic osteomyelitis makes it clear that the progression of the disease is affected by the metabolic and vascular anomalies. Diabetes is commonly known as one of the most significant predisposing conditions especially in diabetic foot infections where secondary soft tissue breakdown and poor healing contribute to the spread of infection to bone [7]. *staphylococcus aureus* is the most important etiological agent in the osteomyelitis in the world, due to its capacity to attache to bone matrix, evade host immunity mechanisms and form biofilms. These attributes lead to resistance to treatment and the outcome is chronic infection, which in most cases necessitates prolonged antimicrobial treatment and recurrent surgery [8]. Infections of biofilms are particularly hard to eliminate and these facts contribute to the significant role of microbiological verification and use of susceptibility-profounded therapy [8]. MRSA was a significant percentage in the current research. Past research indicate that osteomyelitis caused by MRSA can be characterized by more aggressive illnesses, higher surgical care and a longer period of treatment, yet results are determined by the early identification and proper treatment [9]. The use of non-culture-confirmed empirical treatment in the high-antimicrobial-resistance healthcare setting can be a cause of both the failure of treatment and the chronicity of the disease. The findings of histopathology were dominated by the results of chronic inflammatory alterations, death of bone and formation of sequestrum which are characteristic signs of the prolonged osteomyelitis [10]. The results indicate persistent infection, compromised vascularity and poor host clearance, which highlight the need to use a hybrid approach to medicine and surgery that can eradicate microbes and eliminate immune-necrotic tissue. Osteomyelitis is a complex disease that needs a holistic management approach that includes early identification of the disease, thorough microbiological examination and personalised treatment regimen. This is specifically applicable in diabetics and other systemic risks patients to establish multidisciplinary care, which will reduce the chances of complications, amputation risks and overall clinical outcomes [11].

## Conclusion:

Osteomyelitis was the most common problem in this tertiary care cohort, although males had a higher prevalence rate among the middle- aged adults. The occurrence of chronic osteomyelitis had a strong relationship with diabetes mellitus. The causative organism was *staphylococcus aureus* (including methicillin-resistant) and showed a close connection with chronic disease. Chronic inflammatory changes, necrosis and reactive formation of the bone dominated in the histopathological findings. Prompt microbiological testing and antimicrobial treatment are crucial to enhance the results and avoid the development of chronic osteomyelitis in case of early identification of the risk factors.

## Clinical message:

Osteomyelitis is an unresolved issue, especially among diabetic and comorbid patients, because of its chronic and complicated nature. Clinical assessment, imaging, microbiological culture and histopathology are essential to provide accurate and timely diagnosis that can be utilized to manage it. The most common pathogen is *staphylococcus aureus* and MRSA and hence the significance of culture-directed antibiotic therapy is emphasized. In the specified case, surgical intervention is to be focused on comprehensive debridement of necrotic bone and supplemented with the use of suitable antimicrobial treatment. Multidisciplinary approach is an important factor in enhancing the results of the patient and minimizing the chances of chronic or recurrent infection.

## Figures and Tables

**Table 1:** Demographic and clinical features

**Variable**	**Number (%) / Mean ± SD**
Age (years)	39.6 ± 17.2
**Age group (years)**	
0-14	18 (7.7%)
15-30	62 (26.5%)
31-50	94 (40.2%)
>50	60 (25.6%)
**Gender**	
Male	152 (65%)
Female	82 (35%)
**Comorbidities**	
Diabetes mellitus	60 (25.6%)
Peripheral vascular disease	22 (9.4%)
Immunosuppression	10 (4.3%)
None	142 (60.7%)
Symptom duration >6 weeks	112 (47.9%)
Sinus formation	78 (33.3%)

**Table 2:** Association between diabetes mellitus and type of osteomyelitis

**Diabetes Status**	**Acute Osteomyelitis**	**Chronic Osteomyelitis**	**Total**	**p-value**
Diabetic	18	42	60	0.021
Non-diabetic	72	102	174	
Total	90	144	234	

**Table 3:** Microbiological profile

**Isolated Organism**	**Number (%)**
Staphylococcus aureus	102 (43.6%)
Methicillin-resistant *S. aureus*	36 (15.4%)
*Pseudomonas aeruginosa*	28 (12%)
*Escherichia coli*	20 (8.5%)
*Klebsiella species*	12 (5.1%)
*Streptococcus species*	16 (6.8%)
Mixed infections	12 (5.1%)
No growth	8 (3.4%)

**Table 4:** Association between microbiological isolate and type of osteomyelitis

**Organism**	**Acute Osteomyelitis**	**Chronic Osteomyelitis**	**Total**	**p-value**
*Staphylococcus aureus*	36	66	102	0.034
Methicillin-resistant *S. aureus*	8	28	36	
*Pseudomonas aeruginosa*	12	16	28	
Gram-negative others (*E. coli,* *Klebsiella*)	12	20	32	
*Streptococcus species*	6	10	16	
Mixed infections	4	8	12	
No growth	12	8	20	
Total	90	144	234	

**Table 5:** Histopathological findings

**Finding**	**Number (%)**
Acute inflammatory infiltrates	78 (33.3%)
Chronic inflammatory changes	138 (59%)
Necrotic bone	112 (47.9%)
Fibrosis and granulation tissue	98 (41.9%)
Presence of sequestrum	72 (30.8%)
Reactive new bone formation	64 (27.4%)
